# Enhancing arginase 2 expression using target site blockers as a strategy to modulate macrophage phenotype

**DOI:** 10.1016/j.omtn.2022.08.004

**Published:** 2022-08-04

**Authors:** Chiara De Santi, Frances K. Nally, Remsha Afzal, Conor P. Duffy, Stephen Fitzsimons, Stephanie L. Annett, Tracy Robson, Jennifer K. Dowling, Sally-Ann Cryan, Claire E. McCoy

**Affiliations:** 1 School of Pharmacy and Biomolecular Sciences, Royal College of Surgeons in Ireland, D02 YN77 Dublin, Ireland; 2 FutureNeuro SFI Research Centre, Royal College of Surgeons in Ireland, D02 YN77 Dublin, Ireland; 3 Tissue Engineering Research Group, Department of Anatomy and Regenerative Medicine, RCSI University of Medicine and Health Sciences, Dublin, Ireland; 4 SFI Centre for Research in Medical Devices (CÚRAM), RCSI University of Medicine and Health Sciences, Dublin, Ireland; 5 SFI Advanced Materials and Bioengineering Research (AMBER) Centre, RCSI University of Medicine and Health Sciences and Trinity College Dublin, Dublin, Ireland; 6 Trinity Centre for Biomedical Engineering, Trinity College Dublin, Dublin, Ireland

**Keywords:** MT: non-coding RNAs, microRNAs, macrophages, target site blocker, arginase 2, miR-155, PLGA, transfection

## Abstract

Macrophages are plastic cells playing a crucial role in innate immunity. While fundamental in responding to infections, when persistently maintained in a pro-inflammatory state they can initiate and sustain inflammatory diseases. Therefore, a strategy that reprograms pro-inflammatory macrophages toward an anti-inflammatory phenotype could hold therapeutic potential in that context. We have recently shown that arginase 2 (Arg2), a mitochondrial enzyme involved in arginine metabolism, promotes the resolution of inflammation in macrophages and it is targeted by miR-155. Here, we designed and tested a target site blocker (TSB) that specifically interferes and blocks the interaction between miR-155 and *Arg2* mRNA, leading to Arg2 increased expression and activity. In bone marrow-derived macrophages transfected with Arg2 TSB (in the presence or absence of the pro-inflammatory stimulus LPS), we observed an overall shift of the polarization status of macrophages toward an anti-inflammatory phenotype, as shown by significant changes in surface markers (CD80 and CD71), metabolic parameters (mitochondrial oxidative phosphorylation) and cytokines secretion (IL-1β, IL-6, and TNF). Moreover, in an *in vivo* model of LPS-induced acute inflammation, intraperitoneal administration of Arg2 TSB led to an overall decrease in systemic levels of pro-inflammatory cytokines. Overall, this proof-of-concept strategy represent a promising approach to modulating macrophage phenotype.

## Graphical abstract

**Figure:**
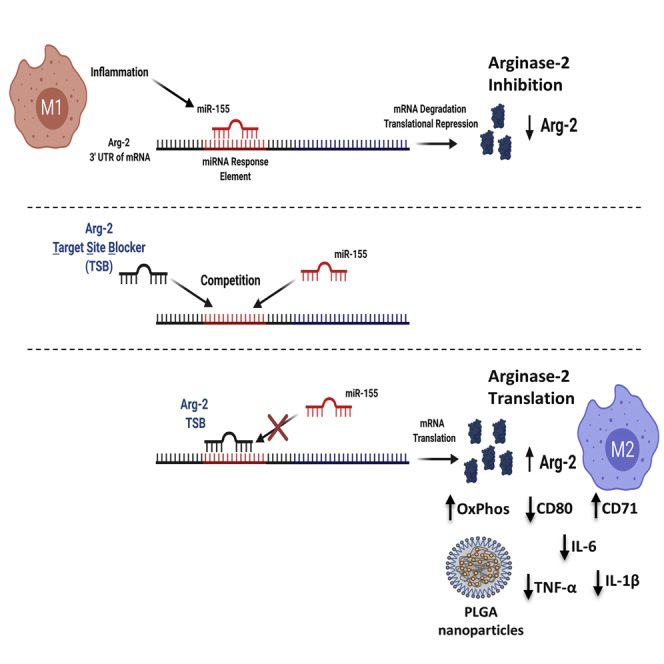

## Teaser

Macrophage reprogramming holds therapeutic potential for inflammatory disorders. Here, we propose a strategy to increase arginase 2, critical for maintaining macrophages in an anti-inflammatory state, by blocking its interaction with miR-155 using a TSB. This led to a shift of the polarization of macrophages toward an anti-inflammatory phenotype.

## Introduction

Macrophages are a sub-type of immune cells that play key roles in the pathogenesis and regulation of the inflammatory reaction. The development of inflammation is critical to fighting infections, and macrophages play a key role in this process by releasing pro-inflammatory cytokines and toxic mediators.^1^ However, if inflammation persists, macrophages can also become destructive, leading to the severe tissue damage observed in variety of chronic inflammatory diseases such as ulcerative colitis,^2^ rheumatoid arthritis,^3^ and multiple sclerosis.^4^ In contrast, macrophages can also produce anti-inflammatory mediators and potentiate cell proliferation, tissue repair, and the healing process.^5^ Therefore, a therapeutic strategy aimed to reprogram pro-inflammatory macrophages toward an anti-inflammatory phenotype could hold merit in the fight against inflammatory diseases.

Arginase 2 (Arg2) is a mitochondrial associated enzyme involved in L-arginine metabolism, hydrolyzing arginine to ornithine and urea.^6^ We have recently shown that Arg2 promotes an anti-inflammatory state in murine macrophages by modulating mitochondrial dynamics, enhancing oxidative phosphorylation (OxPhos) and regulating IL-1β secretion.^7^ Importantly, we have also shown that Arg2 is a target of miR-155 in macrophages.^7^ MicroRNAs (miRNAs) are evolutionarily conserved non-coding RNAs that negatively regulate gene expression by binding to the 3′ untranslated region (UTR) of the target mRNA and repressing translation or decreasing mRNA stability. MiR-155 over-expression is closely associated with various inflammatory disorders and miR-155 inhibition with an anti-miR-155 has shown encouraging results in terms of limiting disease progression in animal models.^8^, ^9^, ^10^, ^11^, ^12^, ^13^, ^14^ However, this strategy could be prone to off-target effects on unintended physiological miR-155 targets, as well as to co-inhibition of other miRNAs.^15^

TSBs are locked-nucleic acid antisense oligonucleotides that specifically compete with miRNAs for the binding to individual miRNA recognition elements (MREs) of a target mRNA, hence preventing them from gaining access to those sites and ultimately leading to increased levels of the target protein. Recently, a TSB targeting the interaction between miR-10a and ligand-dependent nuclear receptor corepressor (*Lcor*) was shown to decrease mitochondrial respiration in macrophages and increase atherosclerotic lesion formation in the aortic arch compared with control TSB.^16^ In non-macrophage related contexts, TSBs have been proposed as therapeutics for cystic fibrosis^17^^,^^18^ and cancer.^19^^,^^20^

In this work, we designed a TSB that specifically blocked the interaction between *Arg2* mRNA and miR-155 (henceforth called Arg2 TSB) in murine macrophages. We assessed whether Arg2 TSB, by increasing the expression of Arg2, could modulate macrophages phenotype in a variety of *in vitro* assays and we further evaluated the effect of Arg2 TSB in an *in vivo* model of acute inflammation.

## Results

### Arg2 TSB effectively blocks miR-155-mediated repression of Arg2

**Figure 1:**
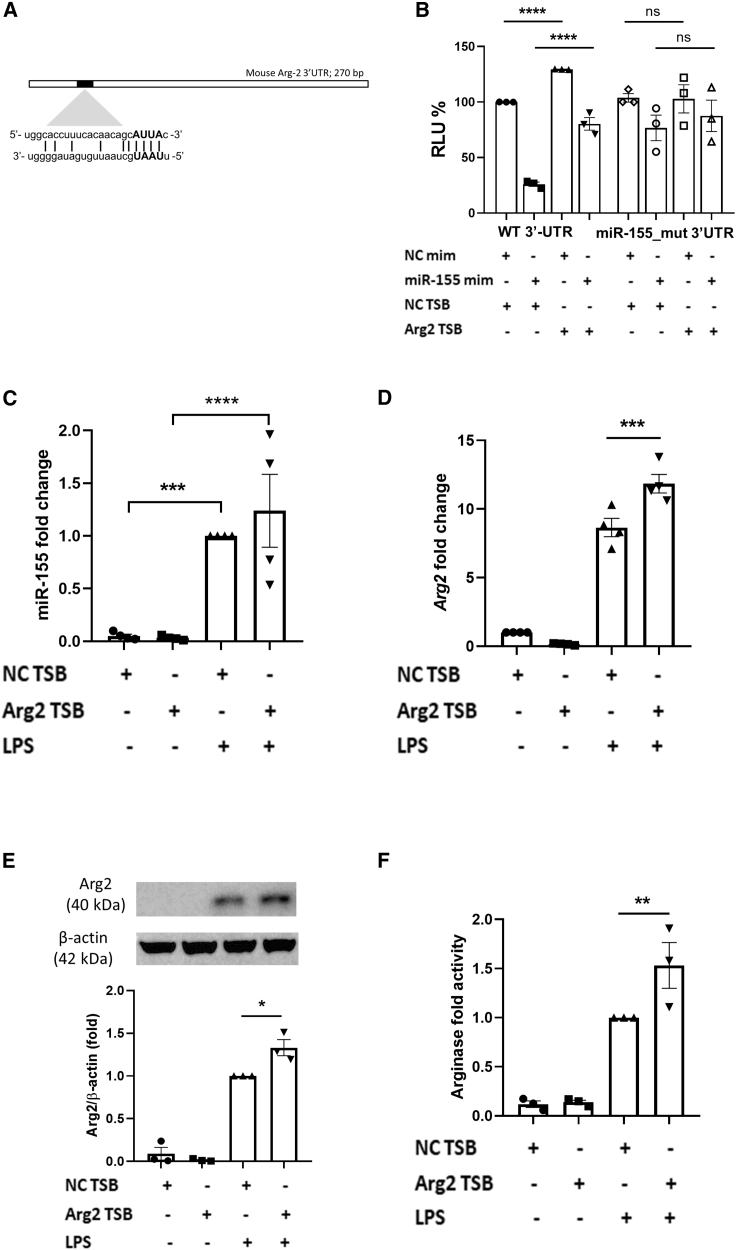
Arg2 TSB effectively blocks miR-155-mediated repression of Arg2 Data are presented as mean ± SEM and were compared by two-way ANOVA (using multiple comparisons test, ∗p ≤ 0.05, ∗∗p < 0.01, ∗∗∗p < 0.001, ∗∗∗∗p < 0.0001). (A) Visual map of the *in silico* predictions of miR-155 binding to its MRE within *Arg2* 3′ UTR. (B) *Arg2* 3′ UTR luciferase activity reported as percent change in relative light units (RLU) in RAW 264.7 cells. Site-directed mutagenesis was applied to pmir_Arg2_wt vector to disrupt the miR-155 MRE. RAW 264.7 cells were co-transfected with wt (first to fourth bars) or miR_155 mutant (fifth to eighth bars) plasmid and TSB and miR mimic (100 ng plasmid, 100 nM TSB, 25 nM mimic, n = 3, in triplicate). Samples co-transfected with WT plasmid, non-targeting control (NC) TSB and NC miR mimic were used as reference and set at 100%. For both WT and miR_155 mutant plasmids, only the comparison between NC and Arg2 TSB (co-transfected with NC or miR-155 mimics) are highlighted in the graph. (C) MiR-155 expression upon stimulation with LPS (100 ng/mL, 24 h) and TSB transfection in primary BMDM (n = 4, in triplicate). LPS-stimulated samples transfected with NC TSB are set as 1. (D) Arg2 TSB effect on endogenous levels of *Arg2* mRNA in primary BMDM (n = 4, in triplicate). Unstimulated samples transfected with NC TSB are set as 1. (E) Arg2 TSB effect on endogenous levels of Arg2 protein in primary BMDM (n = 3, in single). LPS-stimulated samples transfected with NC TSB are set as 1. (F) Arginase activity assay in primary BMDM upon transfection of Arg2 TSB (n = 3, in triplicate). Urea was measured as a byproduct of arginase activity and fold arginase activity was measured by setting LPS-stimulated samples transfected with NC TSB as 1.

To design and test a TSB for specific inhibition of miRNA targets, the MRE within the mRNA of interest needs to be identified and experimentally validated in the appropriate cellular context. *In silico* predictions (TargetScan v7.1) and the previous literature^21^ identified an MRE for miR-155 at position 30–37 of *Arg2* 3′ UTR (Figure 1A). Previous studies have shown that miR-155 mimic can repress luciferase activity in 293T cells when *Arg2* 3′ UTR is cloned into psiCHECK-2 reporter plasmid.^21^ We confirmed this work in macrophages using the RAW 264.7 murine macrophage cell line by illustrating that the over-expression of miR-155 using a miR-155 mimic can inhibit *Arg2* 3′ UTR luciferase activity in a pmir_Arg2_wt plasmid (Figure 1B, first and second bars), an effect that was lost when the miR-155 seed region is mutated (Figure 1B, fifth and sixth bars). We designed a sequence-specific TSB that binds to position 23–38 of *Arg2* 3′ UTR, which should prevent miR-155 from binding and having its desired effect. In Figure 1B, the Arg2 TSB was able to significantly rescue the miR-155-dependent inhibition of pmir_Arg2_wt plasmid (Figure 1B, third and fourth bars, compared with the first and second bars). However, this effect was lost in the mutant plasmid where the miR-155 binding site at position 30–37 was disrupted (Figure 1B, seventh and eighth bars, compared with the fifth and sixth bars), demonstrating that Arg2 TSB specifically blocks the interaction between miR-155 and *Arg2* 3′ UTR through the MRE at position 30–37.

Next, we wanted to evaluate whether Arg2 TSB was able to interfere with the miR-155-dependent regulation of endogenous Arg2 levels in an inflammatory context. MiR-155 up-regulation was achieved *in vitro* by stimulating primary bone marrow-derived macrophages (BMDM) (Figure 1C) and RAW 264.7 murine macrophage cell line (Figure S1A) with lipopolysaccharide (LPS), a Toll-like receptor 4 agonist with a well established ability to potently boost miR-155 expression downstream of NFκB activation in macrophages.^22^ No significant difference in miR-155 expression was observed between negative control (NC) and Arg2 TSB-transfected cells, neither when unstimulated nor when treated with LPS. We then measured the endogenous levels of Arg2 mRNA and protein in BMDM and RAW 264.7 cells after TSB transfection, either in the presence or absence of LPS. Our results show that Arg2 TSB could not boost Arg2 endogenous levels in the absence of LPS. However, in the presence of LPS, a 1.37-fold (p < 0.001) (Figure 1D) and 2.55-fold (p < 0.0001) (Figure S1B) increase in *Arg2* mRNA was observed in Arg2 TSB-transfected BMDM and RAW 264.7 cells, respectively, when compared with NC TSB-transfected cells. This was mirrored at the protein level, where we found Arg2 increases of 1.33-fold (p = 0.029) (Figure 1E) and 1.74-fold (p = 0.015) (Figure S1C) in the same cells.

We next sought to investigate arginase catalytic activity (conversion of L-arginine to L-ornithine) by measuring the by-product urea in TSB-transfected cells. A 1.53-fold (p = 0.0018) and 1.45-fold (p = 0.0046) increase in arginase activity was measured in Arg2 TSB-transfected BMDM (Figure 1F) and RAW 264.7 cells (Figure S1D), respectively, in presence of LPS when compared with NC TSB-transfected cells.

Overall, these results demonstrate that Arg2 TSB can effectively block miR-155-mediated repression of Arg2 by preventing its access to the binding site located at position 30–37 of *Arg2* 3′ UTR, ultimately leading to increased levels of endogenous Arg2 mRNA, protein, and enzymatic activity in LPS-stimulated cells.

### Arg2 TSB modulates surface markers and metabolic parameters in macrophages

We have recently shown that Arg2 plays a pivotal role in resolving a pro-inflammatory phenotype in macrophages.^7^ After confirming that Arg2 TSB was indeed able to boost Arg2 expression in macrophages, we sought to assess whether this increase had an effect on macrophage phenotype in a variety of biological *in vitro* assays.

**Figure 2:**
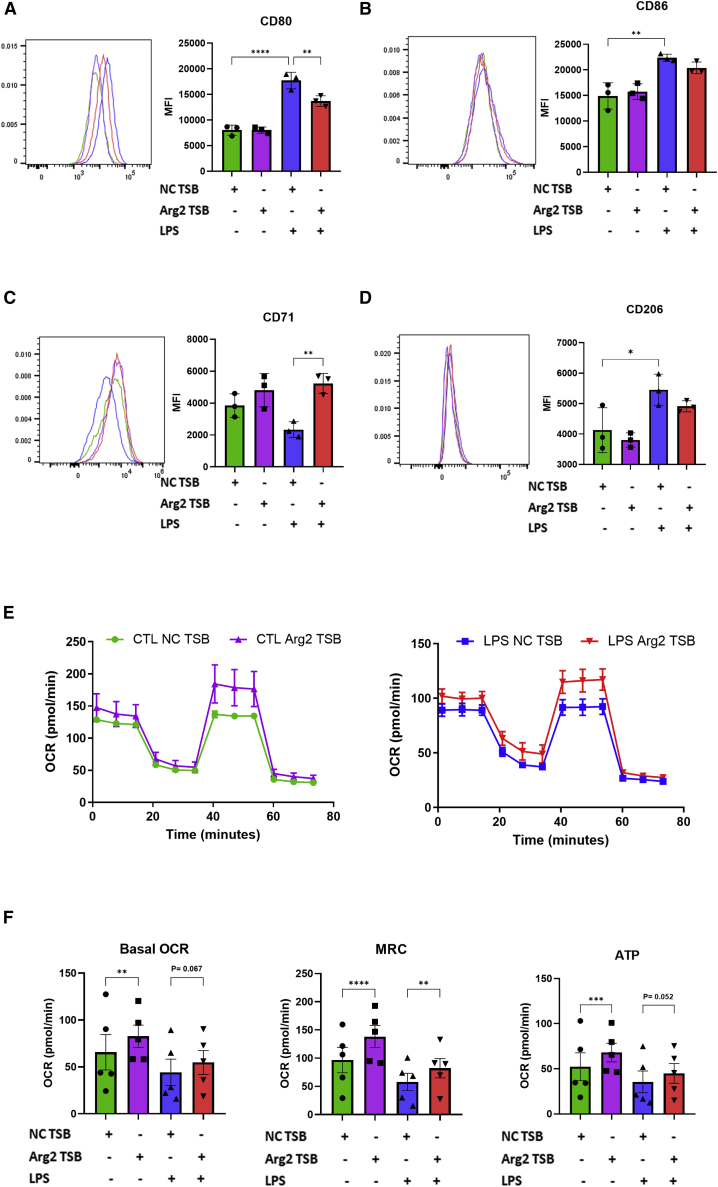
Arg2 TSB modulates surface markers and metabolic parameters in primary BMDM (A–D) Surface markers expression. Pro-inflammatory markers CD80 (A) and CD86 (B) and anti-inflammatory markers CD71 (C) and CD206 (D) surface levels in Arg2 *vs* NC TSB transfected BMDM (in presence and absence of LPS) were compared by flow cytometry (n = 3, in single). Histograms depict the results obtained in one representative experiment. The bar graphs represent the means of mean fluorescence intensity ± SEM of positive cells in three biological replicates. Data were compared by one-way ANOVA (Sidak’s multiple comparisons test, ∗p ≤ 0.05, ∗∗p < 0.01, ∗∗∗∗p < 0.0001). (E) Representative Seahorse metabolic flux trace of BMDM transfected with Arg2 *vs* NC TSB in absence (left) or presence (right) of LPS (10 ng/mL) (n = 5, 4–7 technical replicates). (F) Quantitative oxidative parameters changes in BMDM transfected with Arg2 *vs* NC TSB in absence or presence of LPS (n = 5, 4–7 technical replicates). Data were compared by two-way ANOVA (Tukey’s multiple comparisons test, ∗∗p < 0.01, ∗∗∗p < 0.001, ∗∗∗∗p < 0.0001).

First, we assessed the impact of Arg2 TSB transfection (in absence and presence of LPS) on macrophage polarization by assessing the expression of a panel of pro-inflammatory (CD80 and CD86) and anti-inflammatory (CD71 and CD206) macrophage surface markers by flow cytometry. In the absence of LPS, we did not observe any significant differences in any marker expression. However, in LPS-treated BMDM, Arg2 TSB-transfected cells showed a significant decrease of the pro-inflammatory marker CD80 (Figure 2A) and a significant increase of the anti-inflammatory marker CD71 (Figure 2C) when compared with NC-transfected macrophages. Levels of CD86 were also decreased (but not significantly) upon Arg2 TSB transfection (Figure 2B), while CD206 levels were not changed (Figure 2D).

Next, we assessed the impact of Arg2 TSB transfection on macrophage metabolism. OxPhos is a key metabolic process at the mitochondria that produces energy in aerobic conditions. When macrophages are in a pro-inflammatory stage (e.g., when stimulated with LPS), they decrease their commitment to OxPhos to favor glycolysis by reducing their basal oxygen consumption rates (OCR), maximal respiratory capacity (MRC), and OxPhos-linked ATP production, while an intact OxPhos metabolism is observed in anti-inflammatory macrophages.^23^^,^^24^ We have recently shown OxPhos is enhanced by the presence of Arg2 at the mitochondria.^7^ Therefore, here we investigated whether Arg2 TSB transfection in macrophages would increase OxPhos parameters (OCR, MRC, and ATP production) using metabolic flux analysis in both the absence and presence of LPS. In unstimulated BMDM, we observed that OCR, MRC, and ATP production were significantly increased in Arg2 TSB-transfected cells (Figures 2E and 2F, green *vs* purple line/bar). In cells stimulated with 10 ng/mL LPS for 24 h (Figures 2E and 2F, blue *vs* red line/bar), we observed a significant increase in MRC and an increased trend in basal OCR and ATP production, albeit not statistically significant. We observed very similar results in RAW 264.7 cells upon transfection with TSBs in presence and absence of LPS (Figures S1E and S1F). Overall, this suggests that Arg2 TSB-transfected macrophages boost their oxidative metabolism, even in the presence of a powerful agonist such as LPS, although to a lesser extent than when unstimulated.

### Arg2 TSB encapsulated in biocompatible poly lactic-co-glycolic acid nanoparticles decreases pro-inflammatory cytokines secretion in macrophages

**Figure 3:**
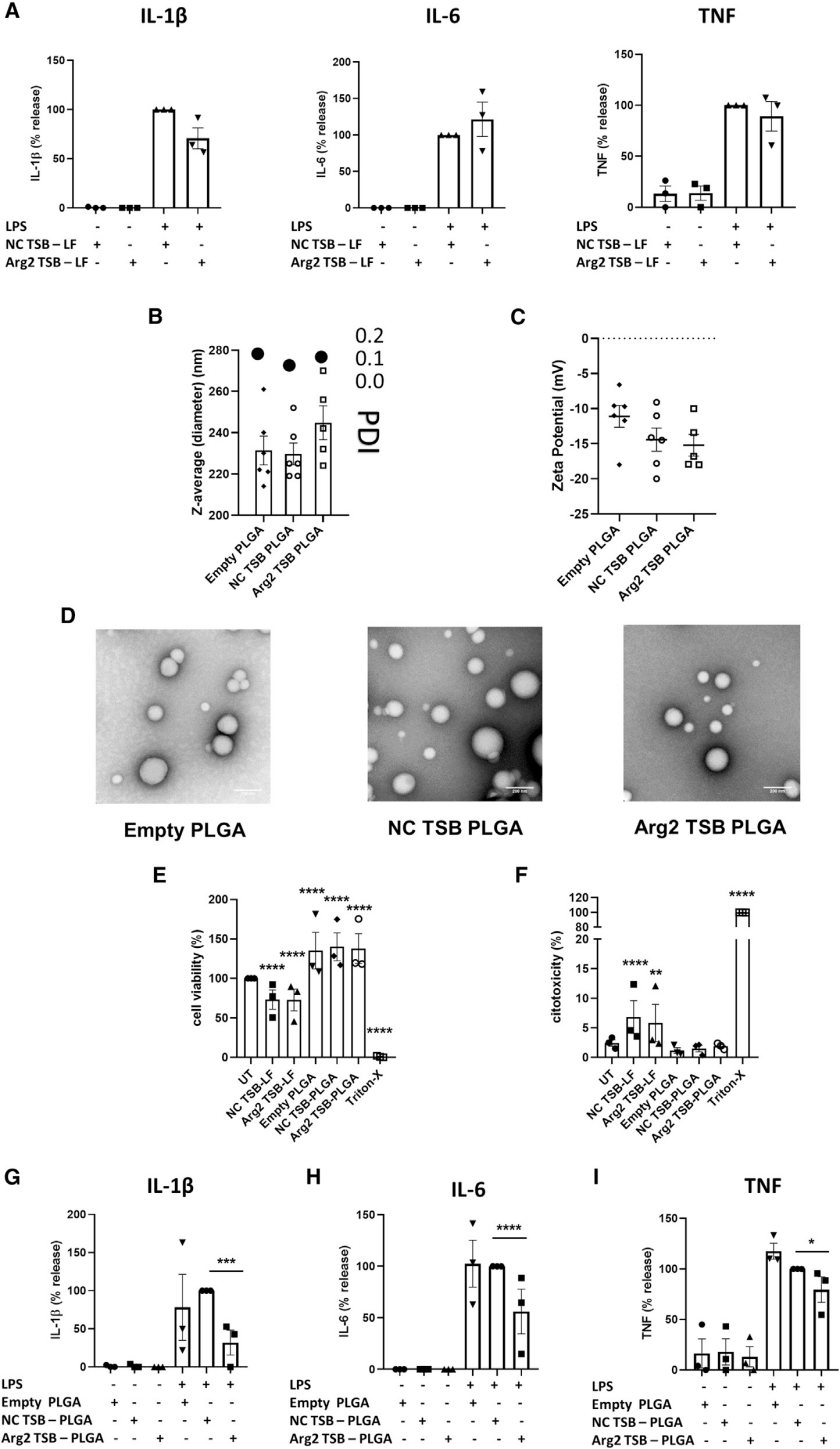
Arg2 TSB encapsulated in biocompatible PLGA NPs decreases pro-inflammatory cytokines secretion in primary BMDM (A) Pro-inflammatory cytokines secretion by BMDM upon transfection of Arg2 *vs* NC TSB using the classical transfection reagent lipofectamine 3000 in presence or absence of LPS (n = 3, in triplicate). Samples transfected with NC TSB were used as reference and set at 100%. (B, C) Physicochemical characterization of empty, NC TSB, and Arg2 TSB PLGA NPs using the Zetasizer system for measuring (B) size (nm) on the left *y* axis (bar charts), poly-dispersity index (PDI) on the right *y* axis (topmost dots) and (C) surface charge (Z-potential, mV). The values represented are the mean averages ± SEM of at least five independent NPs preparation. (D) Representative images of PLGA NPs stained with uranyl acetate replacement stain using transmission electron microscopy (TEM). (E, F) Effect of Arg2 TSB transfection (with lipofectamine 3000 transfection reagent, LF, second and third bars) and PLGA-TSBs NPs (fourth to sixth bars) on primary BMDM (E) viability (3-(4,5-dimethylthiazol-2-yl)-5-(3-carboxymethoxyphenyl)-2-(4-sulfophenyl)-2H-tetrazolium = MTS assay, n = 3, in triplicate) and (F) toxicity (lactate dehydrogenase = LDH assay, n = 3, in triplicate). (G–I) (G) IL-1β, (H) IL-6, and (I) TNF cytokines secretion by BMDM upon transfection of Arg2 *vs* NC TSB encapsulated into PLGA NPs in presence or absence of LPS (n = 3, in triplicate). Samples transfected with NC TSB-PLGA NPs were used as a reference and set at 100%.

We have previously shown that Arg2 can limit IL-1β production in LPS-stimulated macrophages,^7^ suggesting this enzyme could play a role in modulating secretion of pro-inflammatory cytokines in macrophages. We, therefore, investigated the secretion of pro-inflammatory mediators including IL-6, tumor necrosis factor (TNF), and, most important, IL-1β upon TSBs transfection. While we have observed functional effects of Arg2 TSB in BMDM when transfected with classical transfection reagents (i.e., lipofectamine 3000) in flow cytometry and metabolic analyses (Figures 2A–2F), we did not measure any significant changes in cytokine levels when BMDM were transfected with Arg2 *vs* NC TSBs using lipofectamine 3000 (Figure 3A). Therefore, we encapsulated TSBs in poly lactic-co-glycolic acid (PLGA) nanoparticles (NPs) as a proof-of-principle delivery strategy to macrophages where nucleic acid cargo could exert its therapeutic action more efficiently owing to protection from degradation by using a biocompatible polymeric carrier (PLGA). NPs were prepared using PLGA loaded with NC or Arg2 TSB (and a blank empty PLGA). Their diameter was 231.3 ± 6.9, 229.7 ± 5.3, and 244.8 ± 8.3 nm for the empty, NC TSB-, and Arg2 TSB-PLGA NPs, respectively and the polydispersity index value was of approximately 0.1 for all the systems (Figure 3B). All NPs are negatively charged with zeta-potential ranging from −11 mV to −15 mV on average (Figure 3C). Transmission electron microscopy confirmed the size range of approximately 200 nm and showed a round morphology, as expected (Figure 3D). Importantly, unlike transfection with lipofectamine, PLGA NPs did not decrease cell viability or increase cytotoxicity when compared with untransfected macrophages (Figures 3E, 3F for BMDM, S2A and S2B for RAW 264.7 cells).

Our results show that the encapsulation of Arg2 TSB, and hence possibly its greater stability and lower toxicity, is required to obtain a significant decrease in pro-inflammatory mediators in transfected cells *in vitro*. Importantly, we showed that IL-1β secretion is significantly decreased to approximately 32% (p = 0.0009) in BMDM (Figure 3G), when transfected with Arg2 TSB-PLGA compared to NC TSB-PLGA. IL-6 and TNF levels significantly decreased to approximately 56% (p < 0.0001, Figure 3H) and approximately 80% (p = 0.02, Figure 3I) also. Similar effects were also observed in the RAW 264.7 macrophage cell line, where Arg2 TSB transfection, when encapsulated into PLGA NPs, led to significant decrease in IL-1β, IL-6, and TNF (Figure S2C, fifth to tenth bars in each graph). Overall, these data demonstrate that biocompatible PLGA NPs could represent a valuable delivery strategy for modulating secretion of pro-inflammatory cytokines by macrophages.

### Arg2 TSB delivery decreases cytokine secretion in an *in vivo* model of acute inflammation

**Figure 4:**
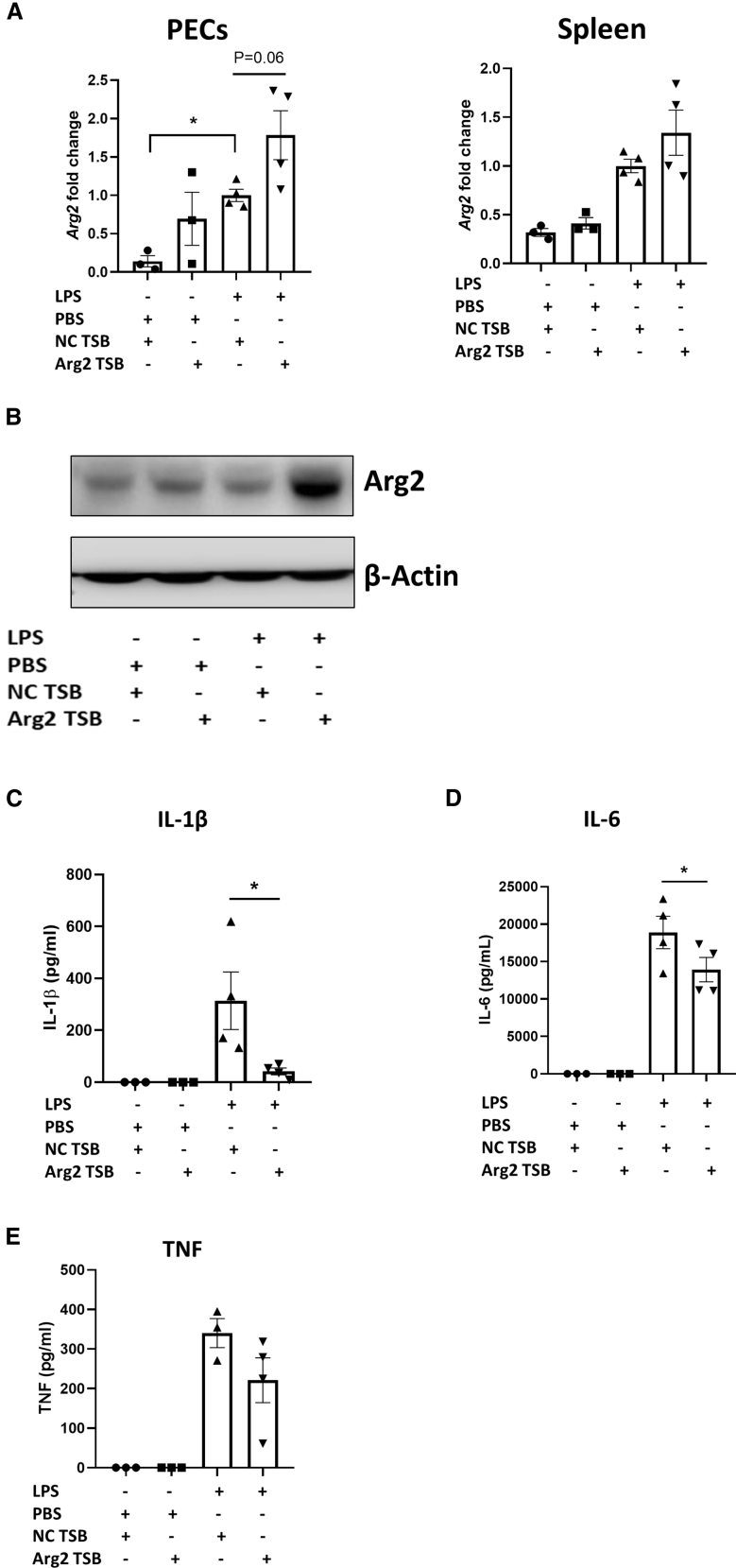
Arg2 TSB delivery decreases pro-inflammatory cytokines secretion in an LPS *in vivo* model WT C57Bl/6J mice were given *i.p*. injection of Arg2 *vs* NC TSB for 24 h followed by LPS at 5 mg/kg (or PBS control) for 8 h. (A) *Arg2* mRNA expression in PECs (left) and spleen (right). Data are presented as mean ± SEM and were compared by one-way ANOVA where mice injected with NC TSB and LPS were set at 1. (B) Arg2 and β-actin in spleen. Blot representative of seven mice from each TSB injection (three of which were then injected with PBS and four with LPS for 8 h). (C) Pro-inflammatory cytokines levels in serum in the LPS model of acute inflammation. Data are presented as mean ± SEM and were compared by one-way ANOVA (Sidak’s multiple comparison test, ∗p < 0.05).

Given these *in vitro* observations, we next sought to test the efficacy of Arg2 TSB in an *in vivo* model of acute inflammation where mice were injected with 200 μg TSB (10 mg/kg dose) and subsequently challenged with an intraperitoneal injection (*i.p*.) of 5 mg/kg LPS for 8 h. We first checked Arg2 TSB ability of enhancing Arg2 expression in the LPS *in vivo* model. Arg2 mRNA (Figure 4A) and protein (Figure 4B) levels were increased in peritoneal exudate cells (PECs) and spleen of mice injected with Arg2 TSB when compared with NC TSB. We next measured the pro-inflammatory mediators IL-6, TNF, and IL-1β to assess whether increasing Arg2 expression through Arg2 TSB could lead to the decrease of these cytokines, similar to what we observed *in vitro*. In serum, Arg2 TSB-injected mice had significantly lower levels of IL-1β (p = 0.01, a decrease from 313.8 ± 110.6 to 42.02 ± 12.97 pg/mL) (Figure 4C) and IL-6 (p = 0.04, a decrease from 18,889 ± 2174 to 13,925 ± 1619 pg/mL) (Figure 4D) when compared with NC TSB-injected mice in the presence of LPS challenge. TNF levels were also decreased, albeit not statistically significantly (Figure 4E). Overall, these *in vivo* data support the hypothesis that a therapeutic strategy aimed to specifically increase Arg2 expression could lead to lower systemic levels of pro-inflammatory mediators that could potentially dampen the overall inflammatory status in a context of acute inflammation.

## Discussion

This work shows that a TSB specifically designed to block the interaction between miR-155 and *Arg2* mRNA can enhance Arg2 expression and modulate its function as a regulator of the inflammatory status of macrophages. Arg2 is a mitochondrial protein involved in arginine metabolism; however, we and other investigators have shown that its role in macrophages goes beyond its enzymatic activity and places Arg2 as a key player in the resolution of the inflammatory response in these innate immune cells.^7^^,^^25^^,^^26^ Therefore, increasing Arg2 levels in macrophages could represent an alternative route for the treatment of inflammatory disorders. The over-expression of proteins could be achieved in multiple ways, for example, by using viral vectors^27^ or by optimizing mechanisms at the post-transcriptional level, including mRNA degradation by miRNAs. Since we and other investigators showed that Arg2 is a target of miR-155,^7^^,^^21^ we developed a proof-of-concept therapeutic strategy whereby a chemically stable and *in vivo* ready TSB was designed to specifically block the interaction between miR-155 and its target *Arg2* mRNA. This has the advantage, compared with more classic approaches like over-expression plasmids and miR-155 inhibitors, of being effective only when miR-155 is expressed in the same cells at the same time as Arg2, thus avoiding widespread off-targets effect (typical of anti-miR strategies owing to the intrinsic ability of miRNAs to regulate multiple mRNAs) and the over-expression of Arg2 when it is not required. Moreover, the TSB will only work in situations when miR-155 expression levels are high, i.e., in cases of inflammation, again, enhancing the efficacy of the TSB to certain conditions and criteria where it is most required.

Once the TSB was designed, we first verified that Arg2 TSB was specific for miR-155 MREs on the *Arg2* 3′ UTR via luciferase assay and effective at increasing Arg2 expression and enzymatic activity in murine primary macrophages and macrophage cell line. Arg2 TSB transfection and the subsequent increase of *Arg2* mRNA did not significantly affect miR-155 expression levels. This was expected, as TSBs do not interact with miRNAs but with their target mRNAs; however, it was important to show given the mounting evidence about a regulatory mechanism called target RNA-directed microRNA degradation (TDMD), whereby high complementarity between miRNA and target mRNAs can actually trigger the degradation of the bound miRNA.^28^, ^29^, ^30^ Our data did not show TDMD happening upon Arg2 TSB transfection, and this is compatible with the literature around TDMD showing that a more extensive pairing through the 3′ region of the miRNA, not present in the interaction between miR-155 and *Arg2* 3′ UTR, seems to be required for it.

Once we had verified the ability of Arg2 TSB to boost Arg2, we assessed whether it was able to recapitulate some of the features we identified as being modulated by Arg2 in murine macrophages. Previously,^7^ we have shown in detail that Arg2 can skew macrophages’ bioenergetics toward an oxidative phenotype by increasing the OxPhos parameters according to metabolic flux analysis. Here we showed that Arg2 TSB can do the same, even in the presence of a potent inflammatory agonist such as LPS. We also knew from our previous study that Arg2 can decrease IL-1β secretion, and here we showed that Arg2 TSB is able to decrease IL-1β levels *in vitro* and *in vivo*. In this study, we also observed a generalized decrease of other pro-inflammatory cytokines such as IL-6 and TNF upon treatment with Arg2 TSB. The biological mechanisms behind this broader effect are unknown; however, Arg2 knock-out mice have previously reported to have higher TNF-α levels compared with wild type (WT) in response to *Helicobacter pylori* infection^25^ and express higher mRNA levels of *IL-1β* as well as *Nos2* (which leads to production and secretion of the pro-inflammatory mediator nitric oxide) in a model of nerve injury,^26^ while the over-expression of Arg2 led to a decrease of inducible nitric oxide protein levels in LPS-stimulated RAW 264.7 cells.^31^ This evidence collectively suggests a pleiotropic role for Arg2 in cytokines secretion and inflammatory signaling. Interestingly, the effect of Arg2 TSB on pro-inflammatory cytokines was not evident (TNF and IL-6) or statistically significant (IL-1β) *in vitro* (BMDM) until we packaged the TSB into biocompatible PLGA NPs. This was not completely unexpected, as a higher efficiency of nano/microparticles loaded with nucleic acids in modulating the expression of pro-inflammatory markers in cultured macrophages when compared with classical transfection reagents have been reported before.^32^^,^^33^ Yet, when injected intraperitoneally *in vivo*, Arg2 TSB significantly decreased IL-1β and IL-6 systemic secretion, suggesting that the chemical formulation/dose of these molecules (which are indeed *in vivo* ready) is sufficient for uptake by macrophages (and potentially other cells) for decreasing pro-inflammatory cytokines in a model of acute inflammation. While not explored in this instance, the functionalization of NPs could be exploited to enhance macrophage specific delivery by the conjugation of ligands or antibodies to target highly expressed surface receptors on these cells^4^ and could provide insightful information on the specific role that macrophages play in the *in vivo* model of LPS-induced inflammation. The targeted delivery of a therapeutic to a particular cell type or disease context is extremely relevant in case of Arg2; while we and others have shown it dampens inflammation in macrophages, Arg2 has been shown to play a primarily pathogenic role in cancer,^34^ atherosclerosis,^35^ and diabetic renal injury.^8^

Furthermore, we aimed to evaluate the ability of Arg2 TSB to modulate surface marker expression as another feature of macrophage polarization and we observed a decrease of CD80 and an increase of CD71 surface expression by flow cytometry in Arg2 TSB-transfected macrophages compared with controls in the presence of an LPS stimulus, while we did not see significant changes in CD86 or CD206 surface levels. CD80 (also called B7-1) is a membrane protein expressed on the surface of various immune cells, including macrophages and dendritic cells, where it is crucial for the activation of the adaptive immune response.^36^ CD80 expression on macrophages can be induced by stimulating them with LPS,^37^ therefore, it is commonly considered as a surface marker for pro-inflammatory macrophages.^38^ CD71 (also known as transferrin receptor protein 1) is a membrane protein required for iron import from transferrin into the cells.^39^^,^^40^ It is up-regulated in the presence of IL-4, and thus has recently been used as a marker for anti-inflammatory polarization in macrophages.^41^^,^^42^ Interestingly, its levels are increased in human rheumatoid patient-derived macrophages in response to miR-155 antagomir delivery.^43^ Our data suggest that, by decreasing a pro-inflammatory and increasing an anti-inflammatory surface marker, Arg2 TSB-transfected macrophages are modulating their polarization toward an anti-inflammatory state. To the best of our knowledge, this is the first report of an effect of Arg2 modulation on surface markers, and although the mechanisms governing this are not understood yet these data further corroborate the involvement of Arg2 in determining the polarization status of macrophages.

There are limitations to this study. While we observed functional effects of Arg2 TSB in multiple assays (flow cytometry, metabolic analysis, cytokine secretion) and could suggest a rationale behind some given our previous investigation,^7^ it was not possible to explore all mechanisms underpinning these functional changes as they were beyond the scope of this work. Ideally, a proteomic study upon transfection of Arg2 TSB could have informed more accurately about the selection of pro- and anti-inflammatory markers to be measured *in vitro* and *in vivo*. We did not test Arg2 TSB when encapsulated in the PLGA NPs in the LPS injury model because the TSBs were *in vivo* ready and the amount of NPs for *in vivo* injection would have required a scale-up of NP preparation which was not feasible. Finally, while this work represents a proof of concept of therapeutic strategy aimed to reprogram macrophages in murine *in vitro* and *in vivo* models, further investigation on the role of Arg2 in human macrophages and its therapeutic potential needs to be carried out.

In conclusion, in this work we have shown that, by blocking the specific interaction between miR-155 and its key targets Arg2 by using a chemically stable TSB, macrophages are able to shift their physiological state away from a pro-inflammatory phenotype toward an anti-inflammatory one. This strategy could be beneficial in the context of diseases mediated by macrophages such as inflammatory, autoimmune or neurological diseases, where it is desirable to reprogram macrophages from a pro-inflammatory toward an anti-inflammatory state as a therapeutic avenue to control inflammation and promote repair.

## Materials and methods

### Cell culture and treatments

All cells were incubated in a humidified incubator at 37°C with 5% CO_2_ levels.

RAW 264.7 murine macrophage cell line was obtained from ATCC and cultured in Dulbecco’s Modified Eagle’s Medium (Sigma, Cat# D5796) supplemented with 10% heat-inactivated fetal bovine serum (FBS) (Sigma, Cat# F9665) and 1% penicillin-streptomycin (pen/strep, 100 U/mL, Sigma, Cat# P4333). Cells were routinely tested to be Mycoplasma negative. Cells were passaged twice a week (1:10) in T75 flasks. All experiments were carried out in early passage numbers, with passage number not exceeding 15 at most.

Bone marrow was isolated from WT C57BL6/J mice 6- to 12-week-old adult female littermates. Mice were euthanized in a CO_2_ chamber and death was confirmed by cervical dislocation. Femurs and tibias were isolated in sterile conditions, and the bone marrow was flushed out using Dulbecco’s PBS. Marrow was spun and incubated with red blood cell lysis buffer (Sigma) to remove red blood cells. A single cell suspension was prepared by passing the cells through a 70-μm cell strainer (Corning). They were then plated in 10-cm petri dishes in complete DMEM supplemented with 10% heat-inactivated FBS and 1% pen/strep. We also added 20% L929-conditioned media to the culture to induce BMDM differentiation, after which cells were incubated for 6 days. In experiments, BMDM were seeded and stimulated in complete DMEM supplemented with 10% L929-conditioned media.

L929 murine fibroblast cell line was obtained by ATCC and maintained in RPMI medium supplemented with 10% FBS and 1% pen/strep. L929 conditioned media was generated from 20 × 10^6^ L929 cells plated in 40 mL complete RPMI-1640 in T175 flasks for 10 days after which the media was filtered and frozen at −20°C until use.

Fresh media was added to the cells before stimulation experiments. LPS (Sigma *Escherichia coli* O111:B4) was diluted from stock concentration of 1 mg/mL in complete DMEM, and used at a final concentration of 100 ng/mL (unless specified otherwise). Cells were typically stimulated for 24 h before conducting further assays.

### Target site-blocker and dual-luciferase assay

TSBs are locked-nucleic acid antisense oligonucleotides that specifically compete with miRNAs for the binding to individual MREs of a target mRNA, hence preventing them from gaining access to those sites. One TSB was designed to compete with miR-155-5p (miR-155) binding to its specific site within the *Arginase-2* (*Arg2*) 3′ UTR (MRE at position 30–37, as predicted by TargetScan v7.1 http://www.targetscan.org/mmu_71/and shown in^21^) and its sequence is GTAATGCTGTTGTGAA (Qiagen, Cat # 339199, ID YT0070992-FDA, MW 5416.27 Da). A scrambled TSB (i.e., not targeting anywhere in the genome, sequence ACGTCTATACGCCCA, Qiagen, Cat# 339199, ID YT0070993-FDA, MW 5016.05 Da) was used as a NC in all experiments.

The full-length (270 bp) murine *Arg2* 3′ UTR was amplified using Q5 High-Fidelity DNA Polymerase (NEB) and inserted into XhoI-digested pmirGLO vector (Promega) downstream to the firefly luciferase (*luc2*) reporter gene using the GenBuilder Cloning Kit (Genscript). Plasmids were isolated from bacterial cultures with the Plasmid Midi Kit (Qiagen, Cat # 12143). To prove the specificity of Arg2-TSB for that particular binding site, a mutagenesis reaction was performed to disrupt its MRE at position 30–37 within the 3′ UTR region using QuikChange Lightning Site-Directed Mutagenesis Kit (Agilent) using the wt plasmid (i.e., pmir_Arg2_wt) as a template. Presence of the mutation in the mutant plasmid (i.e., pmir_Arg2_mut) was subsequently checked by screening with allele-specific oligonucleotide PCR and Sanger sequencing. The sequence of cloning, mutagenesis, and sequencing primers is reported in Table S1.

In luciferase assay experiments, RAW 264.7 cells were seeded in a 96-well plate at a final density of 80,000 cells/well and incubated for 24 h. Cells were then co-transfected with 100 ng pmir_Arg2_wt or pmir_Arg2_mut and 100 nM of Arg2-TSB or NC TSB and 25 nM of miR-155 mimic (ThermoFisher Scientific, Cat# 4464066, ID MC13058) or NC mimic (ThermoFisher Scientific, Cat# 4464058, Negative Control #1). Transfection mixes were prepared in serum-free DMEM using TransIT-X2 Transfection Reagent (Myrus). Luciferase activity was assessed at 24 h after transfection using Dual-Luciferase Reporter Assay (Promega) according to the manufacturer’s instructions. Relative luciferase units expressed as mean value of the firefly luciferase/Renilla luciferase ratio of at least three independent experiments performed in triplicate were used for statistical analyses.

### Gene and protein expression analysis

For gene expression (miR-155 and *Arg2* mRNA), BMDM and RAW 264.7 cells were seeded in a 24-well plate at a final density of 2.5–3.75 × 10^5^ cells/well and after 24 h they were transfected with 100 nM Arg2 TSB or NC TSB in DMEM serum-free medium and lipofectamine 3000 transfection reagent (ThermoFisher Scientific). At 24 h after transfection, cells were stimulated with 100 ng/mL LPS as previously stated for a further 24 h. Total RNA was then extracted using Tri-Reagent (Sigma, Cat# T9424). For miR-155 expression, TaqMan MicroRNA Reverse Transcription kit (ThermoFisher Scientific, Cat# 4366596) and predesigned TaqMan MicroRNA Assays (ThermoFisher Scientific, Cat# 4427975, Assay ID 002571 for mmu-miR-155 and 001973 for U6 snRNA) were used for individual miRNA quantification. For *Arg2* mRNA expression, equal quantities were reverse transcribed into cDNA using High Capacity cDNA reverse transcription kit (ThermoFisher Scientific, Cat# 4368814) following the manufacturer’s protocol. Quantitatie RT-PCR was performed on the 7900 HT and 7500 Real-Time PCR System. Primers for *Arg2* (forward 5′-GGATCCAGAAGGTGATGGAA-3′, reverse 5′-AGAGCTGACAGCAACCCTGT-3′) and three housekeeping genes (*Rplp0*: forward 5-GGACCGCCTGGTTCTCCTAT-3′, reverse 5′-ACGATGTCACTCCAACGAGG-3′; *Tbp*: forward 5′-GAATTGTACCGCAGCTTCAAAAT-3′ and reverse 5′-CAGTTGTCCGTGGCTCTCTT-3′; *Hprt*: forward 5′-GAGGAGTCCTGTTGATGTTGCCAG-3′ and reverse 5′-GGCTGGCCTATAGGCTCATAGTGC-3′) were obtained from Sigma. Expression of miR-155 or *Arg2* relative to U6 snRNA control or housekeeping genes respectively was determined using the 2^(−ΔΔCt)^ method in at least two independent experiments (in triplicate). *Arg2* mRNA levels were also measured in PECs and spleen in the *in vivo* study using the same primers.

For Arg2 protein expression, RAW 264.7 and BMDMs were seeded in a six-well plate at a final density of 1–2 × 10^6^ cells/well and after 24 h they were transfected with 100 nM Arg2 TSB or NC TSB in DMEM serum free medium and lipofectamine 3000 transfection reagent (ThermoFisher Scientific, Cat #L3000008). At 48 h after transfection, cells were stimulated with 100 ng/mL LPS as previously stated for a further 24 h. At least three independent experiments (in single) were performed. Total protein was then extracted using low-stringency lysis buffer (50 mM HEPES [pH 7.5], 100 mM NaCl, 10% glycerol [v/v], 0.5% Nonidet P-40 [v/v], 1 mM EDTA, 1 mM sodium orthovanadate, 0.1 mM PMSF, 1 mg/mL aprotinin, and 1 mg/mL leupeptin). The resulting suspension was centrifuged at 12,000 rpm for 20 min at 4°C, and supernatants were collected and used for SDS-PAGE. Protein samples were normalized by BCA protein assay (Pierce), and denatured by the addition of 4× SDS sample buffer containing 0.2 M DTT and heated at 95°C for 10 min. Equal volumes of whole-cell lysates (5–20 μg) from were separated on 4%–12% Bis-Tris acrylamide gels (ThermoFisher Scientific), transferred to polyvinylidene difluoride membranes (Roche), and probed with mouse anti-Arg2 (1:1000, Invitrogen, Cat# MA5-27815) or anti-β-actin antibodies (1:8000, Sigma, Cat# A5441). Goat anti-mouse IgG, horseradish peroxidase-linked antibody (1:2500, Jackson Immunoresearch, Cat# 115-035-003) and goat anti-mouse IgG Alexa Fluor Plus 488 (1:9000, ThermoFisher Scientific, Cat# A32723) antibody were used as secondary antibodies for 1 h at room temperature (RT) for Arg2 and β-actin primary antibodies, respectively. Detection was achieved using 20× LumiGLO Reagent and 20× Peroxide (Cell Signaling Technology, Cat# 7003) at the Amersham imager for Arg2 blots while fluorescent detection was used for β-actin. For quantitative analysis, the signal intensity of each band was normalized with β-actin densitometry values in at least three independent experiments (in single).

### Arginase assay

Arginase activity was determined in BMDM and RAW 264.7 cells at 48 h after transfection (24 h after LPS stimulation) using the Arginase Activity Assay Kit (Sigma, Cat# MAK112-1KT) following the manufacturer’s protocols in three independent experiments (in triplicate). Briefly, cells were lysed in low-stringency lysis buffer followed by the addition of arginine substrate buffer (provided in the kit) for 2 h at 37°C. Reaction was stopped by addition of urea reagent, and incubated for a further 1 h at RT, after which the absorbance was read at 430 nm to quantify the urea produced against a standard curve.

### Flow cytometry

BMDM were plated in 12-well plates at a final density of 5 × 10^5^ cells/well, transfected with 100 nM Arg2 TSB or NC and stimulated with LPS (10ng/mL) as described above. After 24 h of LPS stimulation, the medium was removed and cells were gently scraped in PBS. Cells were collected by centrifugation at 600×*g*, followed by staining using LIVE/DEAD Fixable Near-IR Dead Cell Stain Kit (1:1000, Invitrogen, Cat #L10119). Cells were washed in fluorescence-activated cell sorting (FACS) buffer (dPBS, 2% FBS, 1 mM EDTA) and incubated with Fc Block for 10 min on ice (1:100, Biolegend, Cat # 101301). Cells were then incubated with antibodies against extracellular antigens F4/80, CD80, CD86, and CD71 for 30 min on ice, followed by two wash steps in FACS buffer. Cells were fixed and permeabilized using Cyto-Fast Fix/Perm Buffer Set (Biolegend, Cat# 426803) followed by intracellular staining for CD206. All data were acquired on the Attune NxT Flow Cytometer (ThermoScientific). The following controls were used: unstained cells, single stained live/dead cells, and full minus one controls or full minus two controls. Single stain compensation controls were generated using Ecomp beads (Invitrogen, Cat# 01–2222) stained with 0.5× antibody concentration used for cell staining. Cells were gated on forward scatter side and side scatter, single cells, and live cells. There were 10,000 live events acquired per sample. RAW FCS files were analyzed using FlowJo Software (FlowJo). Analysis of all markers was performed on the F4/80 positive population. Antibodies listed as follows (fluorochrome, dilution, clone, company, cat number): CD80 (BV421, 1:100, 16–10A1, Biolegend, 104725) CD86 (SuperBright600, 1:160, GL1, Invitrogen, 63-0862-80), F4/80 (Alexa Fluor 488, 1:800, BM8, Biolegend, 123119), CD206 (PercCP/Cyanine5.5, 1:80, C068C2, Biolegend, 141715), CD71 (APC, 1:160, RI7217, Biolegend, 113819).

### Metabolic flux analysis

Cells were plated in 6-well tissue culture plate at a final density of 10^6^ cells/well and after 24 h they were transfected with 100 nM Arg2 TSB or NC TSB in DMEM serum-free medium and lipofectamine 3000 transfection reagent (ThermoFisher Scientific). At 6 (BMDMs)-24 (RAW 264.7) hours after transfection, cells were gently scraped, counted, and re-seeded on an XF 96-well plate (Agilent) in complete DMEM (+10% L929 supernatant in media for BMDM) at a final density of 5 × 10^4^ cells/well. Cells were then stimulated with 10 ng/mL LPS for further 24 h followed by MitoStress test as described in.^7^ Five independent experiments (with multiple wells/treatment) were performed for this assay in BMDM, while preliminary data for one experiments was obtained for RAW 264.7 cells.

### Cytokine measurements

For cytokine measurements, cells were seeded in 96-well plates at a final density of 50–80,000 cells/well. After 24 h, cells were either transfected with Arg2/NC TSB (100 nM) using lipofectamine 3000 as previously stated or with empty PLGA/NC TSB PLGA/Arg2 TSB PLGA NPs resuspended in serum-free DMEM at a NPs concentration of 3.33 mg/mL (typically for each experiment 200 μg of NPs in 600 μL of serum-free DMEM, 100 μL/well) in at least three independent experiments (in triplicate). After 24 h, cells were stimulated with LPS at 100 ng/mL and supernatants removed and analyzed for mouse IL-6, TNF, and IL-1β using an ELISA (DuoSet, R&D, respectively Cat# DY406, DY410 and DY401) according to manufacturers’ instructions. The same ELISA kits were used to measure cytokines in serum collected from the *in vivo* study.

### LPS challenge *in vivo*

Female mice C57BL/6 were housed in the Biomedical Research Facility unit at the Royal College of Surgeons in Ireland. Mice were used at 8–12 weeks of age and maintained according to the regulations of the Health Products Regulatory Authority. This animal study was approved by the RCSI Animal Research Ethics Committee (Ethical approval number 1403) and were performed under the appropriate license (A19127-P045). Animals were randomly categorized into four groups, each receiving a TSB (10 mg/kg) *i.p.* injection followed by LPS (5 mg/kg, *E. coli* 0111:B4, Invivogen) or PBS *i.p.* injection at 24 h after TSB injection: group 1 received NC TSB + PBS (n = 3), group 2 received Arg2 TSB + PBS (n = 3), group 3 received NC TSB + LPS (n = 4), and group 4 received Arg2 TSB + LPS (n = 4). Animals were culled after 8 h after PBS/LPS *i.p.* injection, after which sera, PECS, and spleens were collected for further analyses. PECs were isolated by flushing the peritoneum cavity with PBS containing 5 mM EDTA. Cells were centrifuged and total RNA isolated using the RNeasy Mini kit (Qiagen, Cat# 74106) and stored at −80°C. Spleens were excised, cut in half, snap frozen in liquid nitrogen, and stored at −80°C until time of assay. RNA was extracted from one-half of the spleen by Tri-Reagent extraction and the second half homogenized, assayed for protein quantification by Pierce BCA Protein Assay Kit (ThermoFisher Scientific, Cat # 23227) and western blotting as described above.

### PLGA NPs preparation

Arg2 TSB and NC TSB (Qiagen) were encapsulated in DOTAP/PLGA NPs using the double emulsion solvent evaporation method as previously described.^32^^,^^44^ Briefly, TSBs were condensed with a cationic lipid DOTAP at an N/P (defined as the molar ratio of amine to phosphate groups) ratio of 4:1. Briefly, for each preparation of 50 mg of PLGA NPs, Arg2 (8.1921 μg) and NC (8.09239 μg) TSB were diluted in 200 μL of RNAse-free water and DOTAP (67.62 μg) was dissolved in 200 μL Tert-butanol. The TSB solution was added dropwise to the lipid mixture, mixed, and lyophilized overnight. For empty PLGA NPs, 200 μL RNAse-free water were added to the lipid mixture, mixed, and lyophilized overnight. We dissolved 50 mg PLGA Resomer RG 502 H (Sigma Cat# 719897) in 2.5 mL chloroform and briefly sonicated. Lyophilized TSB/DOTAP was resuspended in RNAse-free water, added to the PLGA solution dropwise with a glass Pasteur pipette and sonicated for a total of three bursts of 5 s in continuous pulses mode at 70% amplitude to form the primary water-in-oil emulsion. The primary emulsion was added dropwise to a 2% poly(vinyl alcohol) (PVA) solution and sonicated on ice for 10 min in continuous pulses mode at 70% amplitude to form a secondary water-in-oil-in-water emulsion and then added to 2% PVA. The emulsion was mechanically stirred in the fume hood overnight to allow the solvent to evaporate and allow NPs formation. NPs were then collected by centrifugation at 20,000×*g* for 15 min at 4°C and washed three times with NaCl 1.13% in deionized water to remove residual PVA. after this, TSB-PLGA NPs were resuspended in RNAse-free water and freeze-dried for 24 h.

### PLGA NPs characterization and morphology

Size and zeta-potential of the TSB-PLGA NPs were measured by dynamic light scattering and by laser Doppler electrophoresis, respectively, on a Zetasizer Nano Series (Malvern Instruments). Measurements were made at RT. PLGA NPs were prepared at a concentration of 0.5 mg/mL in deionized water and 1 mL was used for measurement in the instrument. At least three independent batches of NPs, each prepared in triplicate, were used to determine the size distributions and the surface charge of the PLGA-TSB NPs.

NPs were visualized by transmission electron microscopy to further confirm size and determine the morphology. Briefly, PLGA-TSB NPs were prepared at a concentration of 1 mg/mL in deionized water. We placed 5 μL NPs suspension on a Silicon Monoxide/Formvar coated grid (Mason Technologies). Samples were allowed to air dry for approximately 10–15 min before being negative stained with 2% uranyl acetate alternative solution. Excess stain was removed using filter paper and the grids allowed to air dry fully before analysis. Imaging was performed using a Hitachi H-7650 Transmission Electron Microscope (Hitachi High Technologies, Berkshire, UK) at 120 kV.

### TSB-PLGA NPs effect on macrophage viability

RAW 264.7 cells and BMDM were seeded in 96-well plates at a final density of 40–80,000 cells/well. After 24 h, cells were either left untransfected (control) or transfected with Arg2/NC TSB (100 nM) using lipofectamine 3000 as previously stated or with empty/NC TSB/Arg2 TSB PLGA NPs resuspended in serum-free DMEM at a NPs concentration of 3.33 mg/mL (typically for each experiment 200 μg of NPs in 600 μL serum-free DMEM, 100 μL/well) in three independent experiments (in triplicate). The impact of PLGA NPs on cell viability was assessed using CellTiter 96 Aqueous One Solution Cell Proliferation MTS Assay (Promega, Cat# G3582). To check the cytotoxicity of TSB-PLGA NPs, supernatants were used to measure lactate dehydrogenase release from dying cells using CytoTox 96 Non-Radioactive Cytotoxicity Assay (Promega, Cat# G1780).

### Statistical analyses

Analyses were performed using GraphPad PRISM 8.0. Results are expressed as mean ± SEM and compared as indicated using one-way or two-way ANOVA, as appropriate, followed by Tukey’s, Dunnett’s, or Sidak’s multiple comparisons tests. Differences were considered statistically significant when p ≤ 0.05.

### Data availability statement

All data generated or analyzed during this study are included in this published article (and its supplementary information files).

## Supplemental information

Document S1. Figures S1, S2 and Tables S1

Document S2. Article plus supplemental information

## References

[1] Ross E.A., Devitt A., Johnson J.R. (2021). Macrophages: the good, the bad, and the gluttony. Front. Immunol..

[2] Kałużna A., Olczyk P., Komosińska-Vassev K. (2022). The role of innate and adaptive immune cells in the pathogenesis and development of the inflammatory response in ulcerative colitis. J. Clin. Med..

[3] Jang S., Kwon E.J., Lee J.J. (2022). Rheumatoid arthritis: pathogenic roles of diverse immune cells. Int. J. Mol. Sci..

[4] Nally F.K., De Santi C., McCoy C.E. (2019). Nanomodulation of macrophages in multiple sclerosis. Cells.

[5] Oishi Y., Manabe I. (2018). Macrophages in inflammation, repair and regeneration. Int. Immunol..

[6] S Clemente G., van Waarde A., F Antunes I., Dömling A., H Elsinga P. (2020). Arginase as a potential biomarker of disease progression: a molecular imaging perspective. Int. J. Mol. Sci..

[7] Dowling J.K., Afzal R., Gearing L.J., Cervantes-Silva M.P., Annett S., Davis G.M., De Santi C., Assmann N., Dettmer K., Gough D.J. (2021). Mitochondrial arginase-2 is essential for IL-10 metabolic reprogramming of inflammatory macrophages. Nat. Commun..

[8] Morris S.M., Gao T., Cooper T.K., Kepka-Lenhart D., Awad A.S. (2011). Arginase-2 mediates diabetic renal injury. Diabetes.

[9] Zhang J., Cheng Y., Cui W., Li M., Li B., Guo L. (2014). MicroRNA-155 modulates Th1 and Th17 cell differentiation and is associated with multiple sclerosis and experimental autoimmune encephalomyelitis. J. Neuroimmunol..

[10] Yang Z.B., Chen W.W., Chen H.P., Cai S.X., Lin J.D., Qiu L.Z. (2018). MiR-155 aggravated septic liver injury by oxidative stress-mediated ER stress and mitochondrial dysfunction via targeting Nrf-2. Exp. Mol. Pathol..

[11] Henry R.J., Doran S.J., Barrett J.P., Meadows V.E., Sabirzhanov B., Stoica B.A., Loane D.J., Faden A.I. (2019). Inhibition of miR-155 limits neuroinflammation and improves functional recovery after experimental traumatic brain injury in mice. Neurotherapeutics.

[12] Wan J., Yang X., Ren Y., Li X., Zhu Y., Haddock A.N., Ji B., Xia L., Lu N. (2019). Inhibition of miR-155 reduces impaired autophagy and improves prognosis in an experimental pancreatitis mouse model. Cell Death Dis..

[13] Zhu F., Li H., Liu Y., Tan C., Liu X., Fan H., Wu H., Dong Y., Yu T., Chu S. (2020). miR-155 antagomir protect against DSS-induced colitis in mice through regulating Th17/Treg cell balance by Jarid2/Wnt/β-catenin. Biomed. Pharmacother..

[14] Cao Y.Y., Wang Z., Wang Z.H., Jiang X.G., Lu W.H. (2021). Inhibition of miR-155 alleviates sepsis-induced inflammation and intestinal barrier dysfunction by inactivating NF-κB signaling. Int. Immunopharmacol..

[15] Winkle M., El-Daly S.M., Fabbri M., Calin G.A. (2021). Noncoding RNA therapeutics - challenges and potential solutions. Nat. Rev. Drug Discov..

[16] Wei Y., Corbalán-Campos J., Gurung R., Natarelli L., Zhu M., Exner N., Erhard F., Greulich F., Geißler C., Uhlenhaut N.H. (2018). Dicer in macrophages prevents atherosclerosis by promoting mitochondrial oxidative metabolism. Circulation.

[17] De Santi C., Fernández Fernández E., Gaul R., Vencken S., Glasgow A., Oglesby I.K., Hurley K., Hawkins F., Mitash N., Mu F. (2020). Precise targeting of miRNA sites restores CFTR activity in CF bronchial epithelial cells. Mol. Ther..

[18] Sonneville F., Ruffin M., Coraux C., Rousselet N., Le Rouzic P., Blouquit-Laye S., Corvol H., Tabary O. (2017). MicroRNA-9 downregulates the ANO1 chloride channel and contributes to cystic fibrosis lung pathology. Nat. Commun..

[19] Al-Haidari A.A., Syk I., Thorlacius H. (2017). MiR-155-5p positively regulates CCL17-induced colon cancer cell migration by targeting RhoA. Oncotarget.

[20] Al-Haidari A., Algaber A., Madhi R., Syk I., Thorlacius H. (2018). MiR-155-5p controls colon cancer cell migration via post-transcriptional regulation of Human Antigen R (HuR). Cancer Lett..

[21] Dunand-Sauthier I., Irla M., Carnesecchi S., Seguín-Estévez Q., Vejnar C.E., Zdobnov E.M., Santiago-Raber M.L., Reith W. (2014). Repression of arginase-2 expression in dendritic cells by microRNA-155 is critical for promoting T cell proliferation. J. Immunol..

[22] O'Connell R.M., Taganov K.D., Boldin M.P., Cheng G., Baltimore D. (2007). MicroRNA-155 is induced during the macrophage inflammatory response. Proc. Natl. Acad. Sci. USA.

[23] Kelly B., O'Neill L.A.J. (2015). Metabolic reprogramming in macrophages and dendritic cells in innate immunity. Cell Res..

[24] O'Neill L.A.J., Kishton R.J., Rathmell J. (2016). A guide to immunometabolism for immunologists. Nat. Rev. Immunol..

[25] Hardbower D.M., Asim M., Murray-Stewart T., Casero R.A., Verriere T., Lewis N.D., Chaturvedi R., Piazuelo M.B., Wilson K.T. (2016). Arginase 2 deletion leads to enhanced M1 macrophage activation and upregulated polyamine metabolism in response to Helicobacter pylori infection. Amino acids.

[26] Yin Y., Phạm T.L., Shin J., Shin N., Kang D.W., Lee S.Y., Lee W., Kim C.S., Kim S.R., Hong J., Kim D.W. (2020). Arginase 2 deficiency promotes neuroinflammation and pain behaviors following nerve injury in mice. J. Clin. Med..

[27] Mufarrege E.F., Antuña S., Etcheverrigaray M., Kratje R., Prieto C. (2014). Development of lentiviral vectors for transient and stable protein overexpression in mammalian cells. a new strategy for recombinant human FVIII (rhFVIII) production. Protein Expr. Purif..

[28] Haas G., Cetin S., Messmer M., Chane-Woon-Ming B., Terenzi O., Chicher J., Kuhn L., Hammann P., Pfeffer S. (2016). Identification of factors involved in target RNA-directed microRNA degradation. Nucleic Acids Res..

[29] Fuchs Wightman F., Giono L.E., Fededa J.P., de la Mata M. (2018). Target RNAs strike back on MicroRNAs. Front. Genet..

[30] Kato M. (2018). Target RNA-directed microRNA degradation; which controls which?. Noncoding RNA Investig..

[31] Lee E.J., Lee Y.R., Joo H.K., Cho E.J., Choi S., Sohn K.C., Lee S.D., Park J.B., Jeon B.H. (2013). Arginase II inhibited lipopolysaccharide-induced cell death by regulation of iNOS and Bcl-2 family proteins in macrophages. Mol. Cells.

[32] Kelly C., Yadav A.B., Lawlor C., Nolan K., O'Dwyer J., Greene C.M., McElvaney N.G., Sivadas N., Ramsey J.M., Cryan S.A. (2014). Therapeutic aerosol bioengineering of siRNA for the treatment of inflammatory lung disease by TNFα gene silencing in macrophages. Mol. Pharm..

[33] Huang Y., Guo J., Gui S. (2018). Orally targeted galactosylated chitosan poly(lactic-co-glycolic acid) nanoparticles loaded with TNF-ɑ siRNA provide a novel strategy for the experimental treatment of ulcerative colitis. Eur. J. Pharm. Sci..

[34] Pilanc P., Wojnicki K., Roura A.J., Cyranowski S., Ellert-Miklaszewska A., Ochocka N., Gielniewski B., Grzybowski M.M., Błaszczyk R., Stańczak P.S. (2021). A novel oral arginase 1/2 inhibitor enhances the antitumor effect of PD-1 inhibition in murine experimental gliomas by altering the immunosuppressive environment. Front. Oncol..

[35] Khallou-Laschet J., Varthaman A., Fornasa G., Compain C., Gaston A.T., Clement M., Dussiot M., Levillain O., Graff-Dubois S., Nicoletti A., Caligiuri G. (2010). Macrophage plasticity in experimental atherosclerosis. PLoS One.

[36] Mondino A., Jenkins M.K. (1994). Surface proteins involved in T cell costimulation. J. Leukoc. Biol..

[37] Schmittel A., Scheibenbogen C., Keilholz U. (1995). Lipopolysaccharide effectively up-regulates B7-1 (CD80) expression and costimulatory function of human monocytes. Scand. J. Immunol..

[38] Raggi F., Pelassa S., Pierobon D., Penco F., Gattorno M., Novelli F., Eva A., Varesio L., Giovarelli M., Bosco M.C. (2017). Regulation of human macrophage M1-M2 polarization balance by hypoxia and the triggering receptor expressed on myeloid cells-1. Front. Immunol..

[39] Kawabata H. (2019). Transferrin and transferrin receptors update. Free Radic. Biol. Med..

[40] Allden S.J., Ogger P.P., Ghai P., McErlean P., Hewitt R., Toshner R., Walker S.A., Saunders P., Kingston S., Molyneaux P.L. (2019). The transferrin receptor CD71 delineates functionally distinct airway macrophage subsets during idiopathic pulmonary fibrosis. Am. J. Respir. Crit. Care Med..

[41] Van den Bossche J., Baardman J., Otto N.A., van der Velden S., Neele A.E., van den Berg S.M., Luque-Martin R., Chen H.J., Boshuizen M.C.S., Ahmed M. (2016). Mitochondrial dysfunction prevents repolarization of inflammatory macrophages. Cell Rep..

[42] Minhas P.S., Latif-Hernandez A., McReynolds M.R., Durairaj A.S., Wang Q., Rubin A., Joshi A.U., He J.Q., Gauba E., Liu L. (2021). Restoring metabolism of myeloid cells reverses cognitive decline in ageing. Nature.

[43] Paoletti A., Rohmer J., Ly B., Pascaud J., Rivière E., Seror R., Le Goff B., Nocturne G., Mariette X. (2019). Monocyte/macrophage abnormalities specific to rheumatoid arthritis are linked to miR-155 and are differentially modulated by different TNF inhibitors. J. Immunol..

[44] Fernández Fernández E., Santos-Carballal B., de Santi C., Ramsey J.M., MacLoughlin R., Cryan S.A., Greene C.M. (2018). Biopolymer-based nanoparticles for cystic fibrosis lung gene therapy studies. Materials.

