# Supplementary material for: Enhancing arginase 2 expression using target site blockers as a strategy to modulate macrophage phenotype
Source: Mol Ther Nucleic Acids. 2022 Aug 4;29:643–55. doi: 10.1016/j.omtn.2022.08.004 (PMC9424864; doi:10.1016/j.omtn.2022.08.004)
Supplement: Document S1. Figures S1, S2 and Tables S1 [file mmc1.pdf]

OMTN, Volume *29*

Supplemental information

Enhancing arginase 2 expression using target site blockers as a strategy to modulate macrophage phenotype

Chiara De Santi, Frances K. Nally, Remsha Afzal, Conor P. Duffy, Stephen Fitzsimons, Stephanie L. Annett, Tracy Robson, Jennifer K. Dowling, Sally-Ann Cryan, and Claire E. McCoy

Supplementary Tables

Supplementary Table 1. Primers names and sequences employed in the cloning and mutagenesis of pmir_Arg2_wt/mut. Mutagenesis primers: the mutant nucleotides are reported in capital letter, bold. ASO-PCR primers: Allele-Specific Oligonucleotide primers (wild type and mutant nucleotides in capital letter, bold) were designed to screen mutant from non-mutant colonies after mutagenesis. ASO-forward primers were used in combination with pmir_seq_R. Sequencing primers: pmir_seq_F and pmir_seq_R primers were designed on the plasmid sequence and they were employed for post-cloning screening and sequencing check.

Name	Sequence
**Cloning primers**	
Arg2_clon_F	aacgagctcgctagcctcgaggaaatactgtactctggcac
Arg2_clon_R	caggtcgactctagactcgagtatgatatactaaggtaataaatg
**Mutagenesis primers**	
Arg2_TSB_MUT_F	ctctggcacctttcacaacagc**TAAT**cagagttgcaaggcattcgaag
Arg2_TSB_MUT_R	cttcgaatgccttgcaactctg**ATTA**gctgttgtgaaaggtgccagag
**ASO-PCR primers**	
ASO_Arg2_wt	cacctttcacaacagc**ATTA**
ASO_Arg2_mut	cacctttcacaacagc**TAAT**
**Sequencing primers**	
pmir_seq_F	gtggtgttgtgttcgtggac
pmir_seq_R	cagccaactcagcttccttt

Supplementary Figures

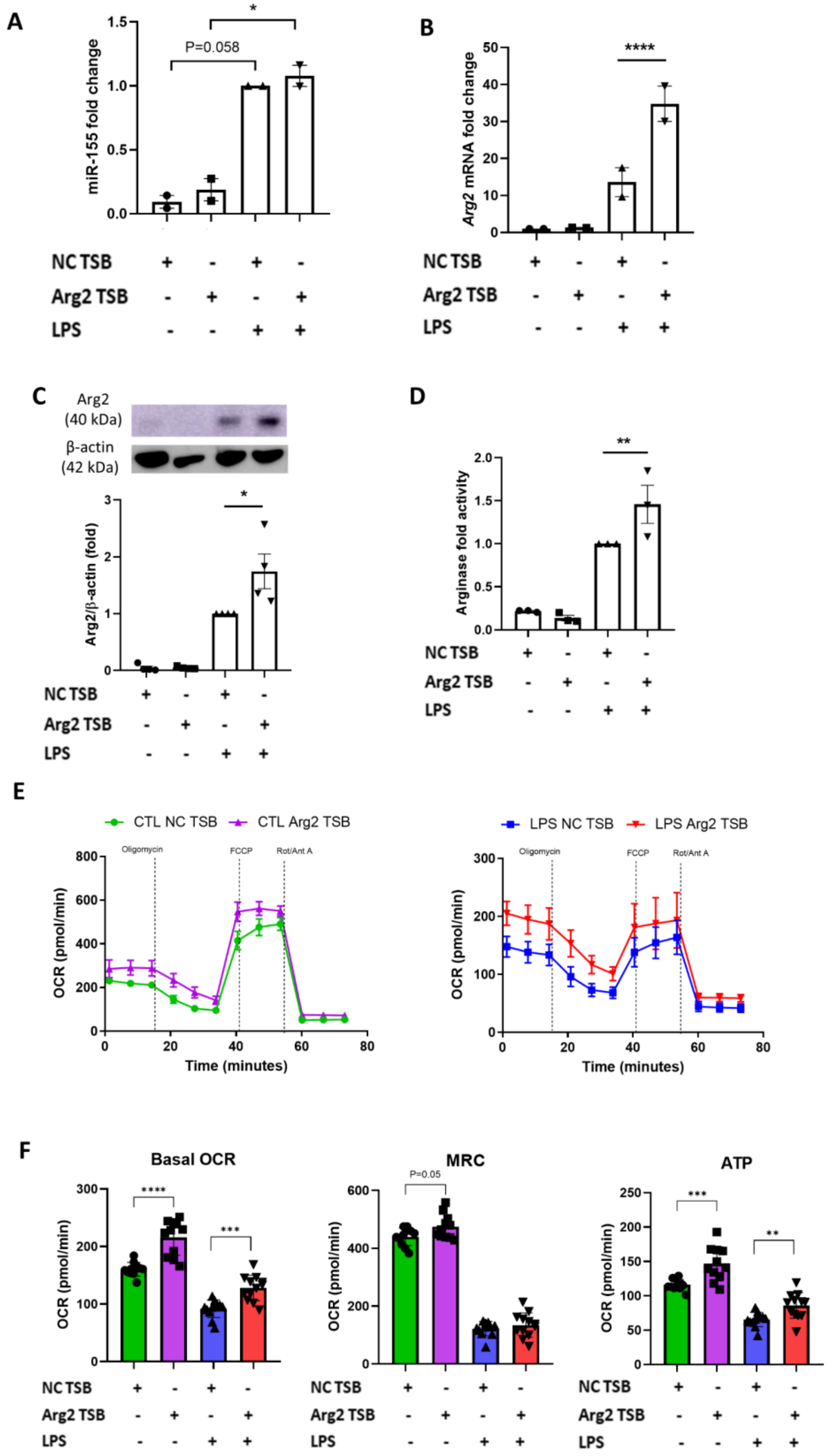

Supplementary Figure 1. Arg2 TSB transfection in RAW 264.7 murine macrophage cell line. (A) MiR-155 expression upon stimulation with LPS (100 ng/ml, 24 hours) and TSB transfection in RAW 264.7 cells (n=2, in triplicate). LPS-stimulated samples transfected with NC TSB are set as 1. (B) Arg2 TSB effect on endogenous levels of *Arg2* mRNA in RAW 264.7 cells (n=2, in triplicate). Unstimulated samples transfected with NC TSB are set as 1. (C) Arg2 TSB effect on endogenous levels of Arg2 protein in RAW 264.7 cells (n=4, in single). LPS-stimulated samples transfected with NC TSB are set as 1. (D) Arginase activity assay in primary RAWs 264.7 upon transfection of Arg2 TSB (n=3, in triplicate). Urea was measured as by-product of arginase activity and fold arginase activity was measured by setting LPS-stimulated samples transfected with NC TSB as 1. (E) Seahorse metabolic flux trace of RAW 264.7 cells transfected with Arg2 vs NC TSB in absence (left panel) or presence (right panel) of LPS (10ng/ml) (n=1, 11-12 technical replicates). (F) Quantitative oxidative parameters changes in RAW 264.7 cells transfected with Arg2 vs NC TSB in absence or presence of LPS (n=1, 11-12 technical replicates). Data were compared by one-way ANOVA (Sidak's multiple comparisons test, **$p<0.01$, ***$P<0.001$, ****$p < 0.0001$).

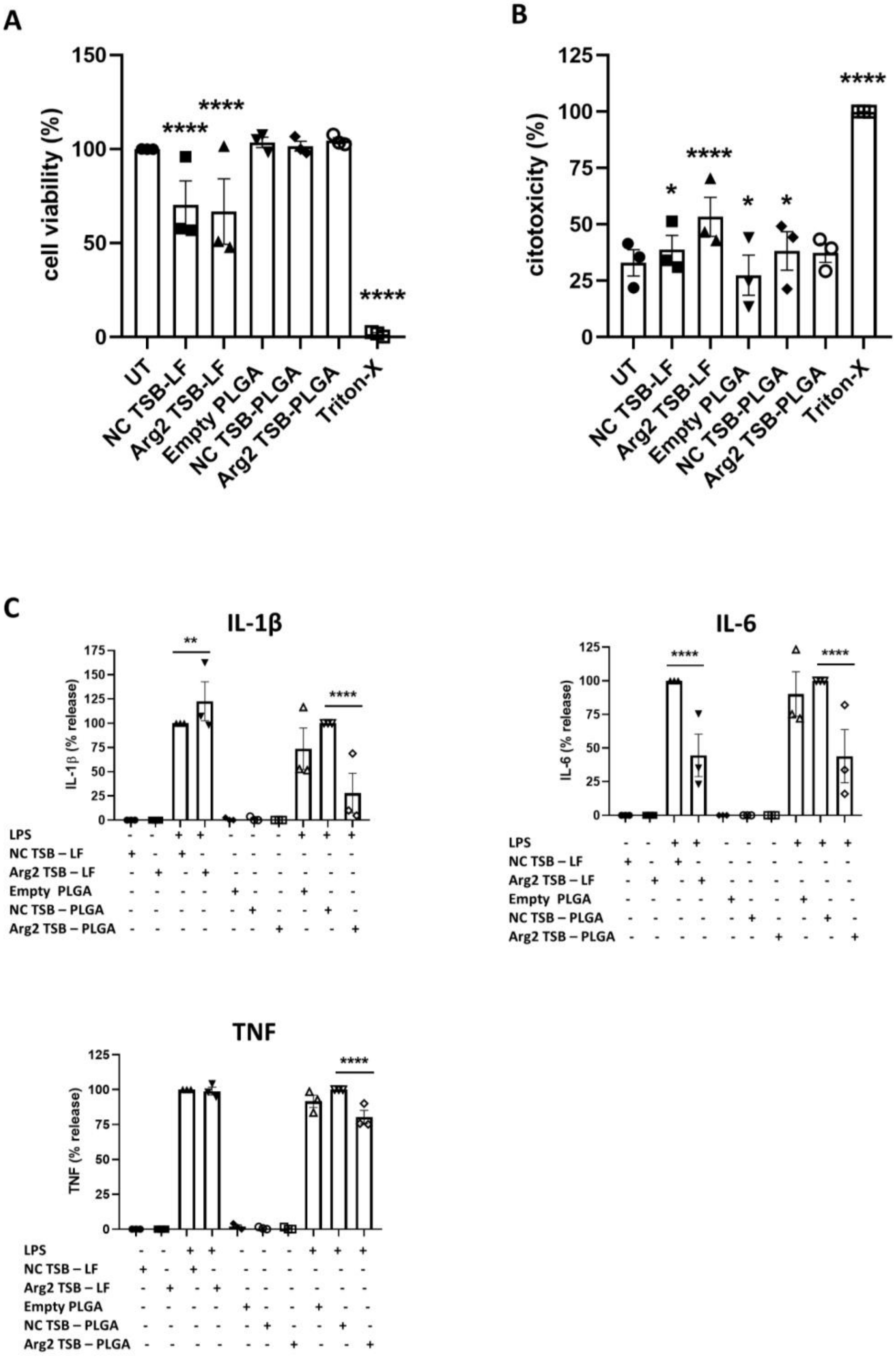

Supplementary Figure 2. Arg2 TSB encapsulated in biocompatible PLGA nanoparticles in RAW 264.7 murine macrophage cell line. (A-B) Effect of Arg2 TSB transfection (with lipofectamine 3000 transfection reagent, LF, 2nd and 3rd bars) and PLGA-TSBs nanoparticles (NPs) (4th-6th bar) on RAW 264.7 cells (A) viability (3-(4,5-dimethylthiazol-2-yl)-5-(3-

carboxymethoxyphenyl)-2-(4-sulfophenyl)-2H-tetrazolium = MTS assay, n=3, in triplicate) and (B) toxicity (lactate dehydrogenase = LDH assay, n=3, in triplicate). UT=untreated, Triton-X was used as positive control of cell death. (C) Pro-inflammatory cytokines secretion by RAW 264.7 cells upon transfection of Arg2 vs NC TSB either using the classical transfection reagent lipofectamine 3000 (LF, 1st-4th bar) or encapsulated into PLGA NPs (5th-10th bar) in presence or absence of LPS (n=3, in triplicate). Samples transfected with NC TSB (either 'naked' or encapsulated into PLGA NPs) were used as reference and set at 100%.
